# Does having siblings really protect against childhood atopic diseases? A total population and within-family analysis

**DOI:** 10.1007/s10654-024-01104-w

**Published:** 2024-02-06

**Authors:** Juha Luukkonen, Heta Moustgaard, Pekka Martikainen, Hanna Remes

**Affiliations:** 1 Population Research Unit, University of Helsinki, Helsinki, Finland; 2 Max Planck–University of Helsinki Center for Social Inequalities in Population Health, Helsinki, Finland; 3 Helsinki Institute for Social Sciences and Humanities, University of Helsinki, Helsinki, Finland; 4 Max Planck Institute for Demographic Research, Rostock, Germany

**Keywords:** Microbiota hypothesis, Atopic disease, Allergy, Asthma, Atopic eczema, Medication, Birth order, Family size, Fixed effects

## Abstract

The association between having older siblings and decreased risk for atopic symptoms is well-established. This has been interpreted as evidence for the microbiota hypothesis, i.e. that increased early-childhood microbial exposure caused by siblings protects from immune hypersensitivities. However, possible confounders of the association have received little attention. We used register data on Finnish cohorts born in 1995–2004 (*N* = 559,077) to assess medication purchases for atopic diseases: antihistamines, eczema medication, asthma medication and Epinephrine. We modelled the probability of atopic medication purchases at ages 0–15 by birth order controlling for important observed confounders and all unobserved genetic and environmental characteristics shared by siblings in a within-family fixed effects model. We further studied medication purchases among first-borns according to the age difference with younger siblings to assess whether having younger siblings in early childhood is beneficial. Having older siblings was associated with a lower probability of atopic medication purchases. Compared to first-borns, the probability was 10–20% lower among second-borns, 20–40% lower among third-borns, and 30–70% lower among subsequent children, depending on medication type. Confounding accounted for up to 75% of these differences, particularly for asthma and eczema medication, but significant differences by birth order remained across all medication types. Among first-borns, a smaller age difference with younger siblings was related to a lower likelihood of atopic medication use. Our results, based on designs that account for unobserved confounding, show that exposure to siblings in early childhood, protects from atopic diseases, and thus strongly support the microbiota hypothesis.

**Supplementary Information:**

The online version contains supplementary material available at 10.1007/s10654-024-01104-w.

## Introduction

The prevalence of childhood atopic diseases has increased globally in the past decades [1, 2] and their public health burden is substantial [2, 3]. While atopic diseases are influenced by both genetic and environmental risk factors, the rapidly increased prevalence is likely due to changes in environmental exposures [3]. According to the so-called microbiota hypothesis, exposure to diverse micro-organisms boosts the development of the immune system and this development is compromised by less diverse microbiota in the immediate environment, resulting in inflammatory responses to otherwise harmless allergens [3, 4].

Strachan (1989) laid the foundations for this hypothesis suggesting that increased microbial exposure in childhood, brought about by siblings, protected from developing immune hypersensitivities [5]. The relationship between having siblings and atopic diseases has since been extensively studied with consistent findings of an association between a larger number of siblings and decreased atopy risk [6, 7]. As the first year of life has been found to be most important for the development of the immune system [8], birth order and timing of the exposure to siblings are also likely to make a difference. For those with an older sibling, the exposure starts from birth, and the higher the birth order, the more siblings there are to affect the early microbial exposure. In line with this hypothesis, higher birth order has been found to be protective of atopic diseases [7]. Having older siblings has also been associated with more diverse gut and airway microbiota in early childhood [9–11], and in turn, diverse microbiota has been associated with less atopic diseases [12, 13]. However, an explicit assessment on how birth order and number of siblings jointly associate with the risk of childhood atopic diseases is still lacking.

Another main shortcoming of the prior literature is the neglect of possible unobserved confounding. Both prenatal and postnatal environmental exposures are likely to differ by birth order, potentially leading to bias. For example, according to the so-called in utero programming hypothesis, lower rates of atopic diseases among younger siblings may relate to decreased prenatal immunoglobulin E level in higher-order pregnancies [14]. Differences in the mode of delivery may also confound the associations: Caesarean sections, that are less common in higher-order pregnancies, are associated with increased risk of some atopic diseases [15]. Regarding the postnatal environment, higher birth order implies larger family size, which may be associated with important socioeconomic and environmental risk factors for atopic diseases. For example, larger families tend to live in more rural and biodiverse environments, exposures that are associated with lower risk for atopic diseases [16–18].

We aim to alleviate the shortcomings in previous research by quantifying the associations of birth order within families of different sizes in models that extensively control for confounding by observed family characteristics. Next, we assess whether birth order is associated with atopic diseases when additionally controlling for confounding by all unobserved genetic and environmental characteristics shared by siblings with a within-family fixed effects design. To our knowledge, this is the first study to use a within-family design to assess the association between birth order and atopic diseases.

Finally, to further investigate the importance of the timing of sibling exposure, and whether the first-born children may still benefit from having younger siblings, we assess the atopic disease risk of first-born children according to their age difference to the second-born sibling. This also allows us to address the microbiota hypothesis without any birth-order-related confounding in prenatal exposures, such as immunoglobulin E levels [14]. To our knowledge, only one study has previously assessed the age difference of siblings, finding that a smaller age difference is a strong predictor of a richer airway and gut microbiota by age six, over and above the number of siblings [10]. However, no evidence on treated atopic diseases are currently available.

## Data

The study population consisted of children born in 1995–2004 identified from the longitudinal population register of Statistics Finland. This individual-level register contains annual information on the full population residing in Finland with linkages between biological family members, allowing identification of parents and full siblings. The data were linked with individual-level information on medication purchases from the national prescription register maintained by the Social Insurance Institution of Finland [19], as well as information on the mode of delivery from the Medical Birth Register.

We included individuals present throughout ages 0–15 (*N* = 559,077), excluding those who emigrated (*n* = 20,015) or died (*n* = 3,275). In the within-family analyses of birth order, we restricted the sample to children with at least one full sibling to compare with (*N* = 324,306) and in the age-difference analyses we only included first-borns (*N* = 266,876).

### Outcome: atopic medication purchases

We used reimbursed purchases of prescription medications as measures for common childhood atopic diseases: allergic rhinitis, allergic eczema, asthma, and severe allergic reactions. All residents of Finland are entitled to partial reimbursement for prescription-issued medication, provided directly at pharmacies [19]. The medications were identified and categorised according to the Anatomic Therapeutic Chemical (ATC) classification (Table 1).

**Table 1:** Medication measuring different atopic diseases

Term in this study	Atopic disease symptoms	Medication	ATC-codes
Antihistamines	Allergic reaction; allergic rhinitis	Antihistamines for systemic use.	R06
Eczema medication	Atopic eczema; allergic dermatitis	Corticosteroids for topical use.	D07
Asthma medication	Asthma	Inhaled corticosteroids and combination inhalers.	R03BA01-08; R03BB04; R03BC01; R03BC03; R3DA05; R03DC01; R03DC03; R03AK03-13; R03AL02
Epinephrine	Severe allergic reaction; anaphylaxis	Epinephrine injectors.	C01CA24

Not all medications used for atopic diseases were captured by our data, as purchases of over-the-counter antihistamines and eczema medication as well as few of the prescription-only products are not reimbursed. However, there is a clear incentive to obtain a prescription and reimbursement in cases of long-term need, or need of more potent medication. All asthma medication require a prescription and are reimbursed [20]. In sensitivity analyses, we also used special reimbursement rights that are granted for individuals with chronic asthma confirmed with a medical evaluation including pulmonary function tests [21] as an additional measure for asthma. All Epinephrine medication also require a prescription, and a vast majority of products are reimbursed [20].

We measured purchases of each medication type with an indicator for at least one purchase by the end of the calendar year the child turns 15 years. In sensitivity analyses we used higher cut-points of having atopic medication purchases in > 1, >2 and > 3 years between ages 0–15.

### Exposures: birth order and age difference

Sibling information was based on biological full siblings. In 2018, roughly 10% of Finnish children lived in families with half or step siblings [22]. As our measurement ignores the presence of other than full siblings, we also ran a sensitivity analysis among children who lived with both their biological parents without any step- or half-siblings between ages 0–15.

#### Birth order and family size

Birth order was classified as 1, 2, 3, 4+. Family size measured the number of biological children of the same parents, classified as 1 (only child), 2, 3, 4+. To assess birth order in families of different sizes, we also constructed a categorical variable measuring both birth order and family size: only child (#1/1), first-born of two siblings (#1/2), second-born of two siblings (#2/2) and so forth until fourth-born or later of four or more biological children in the family. Both family size and birth order were based on information on all siblings born alive by the time the index child was 15 years old.

#### First-born child’s age difference with second-born sibling

The age difference between the first and second-born siblings was rounded to the nearest full year with categories ranging from one to five or more years. We also included separate categories for twins, with no age difference, and only children. It should be noted that twins have an increased asthma risk due to shorter gestational age [23, 24], but to our knowledge, a similar relationship has not been documented with other atopic diseases. As a sensitivity analysis, we also assessed the second-born child’s age difference to their third-born sibling.

### Observed confounders

We controlled for a number of important observed confounders measured in the year of birth unless otherwise specified. Model 1 controlled for sex and birth year of the child, and parental immigrant status (at least one parent born abroad), all important risk factors for atopic diseases [25].

In Model 2, we added controls for characteristics of the child’s place of residence at time of birth. Geographical area dummies (NUTS3 regions) controlled for potential regional differences in access to healthcare and prescription practices. We also controlled for the level of urbanicity, as less biodiverse and more polluted urban environments have been associated with increased rates of asthma and allergies as opposed to rural environments [16, 17]. This urban-rural classification by the Finnish Environment Institute is based on a 250 × 250 m grid that takes into account population density, building density and development and land use. It consists of seven categories spanning from inner city to sparsely populated countryside [26].

In Model 3, we added controls for household socioeconomic position: highest parental education (basic, secondary, or tertiary) and household income decile based on annual disposable income per household consumption units. These variables control for possible socioeconomic differences in the quality of housing, treatment seeking behaviour and affordability of medication.

Finally, in Model 4, we further controlled for the mode of delivery (Caesarean section vs. other), to address the higher likelihood of Caesarean sections among first-born children. We also controlled for parental atopic medication purchases (no vs. at least one purchase between offspring ages 0–15) as separate dummies for each medication type to address genetic liability for atopic diseases. Parents with experience of atopic diseases may also be more likely to seek treatment for their children.

## Methods

We used linear probability models with heteroscedasticity robust standard errors to predict the probability of atopic medication purchases by the age of 15. When predicting medication use by birth order and family size among the full population we controlled for observed confounders in four hierarchical models as described above.

Further, to control for unmeasured familial confounding, we then assessed birth order separately using the same hierarchical models and sibling fixed-effects models which are based on comparisons of full siblings and thus control for all exposures shared by siblings. For these birth-order models, we only included children with at least one full sibling and also added a control for family size (Models 1b, 2–5).

Finally, we predicted medication purchases among first-born children by the age difference to their second-born sibling to assess whether less time spent as the only child decreases the probability of atopic diseases. Sibling fixed effects were not applicable for this analysis, but besides controlling for the observed confounders, we used cousin fixed effects as a weaker control for familial confounding by comparing maternal cousins.

## Results

The prevalence of atopic medication in childhood varied from 37.2% for antihistamines to 2.8% for Epinephrine (Table 2, panel A). The prevalence of all atopic medication types decreased notably with higher birth order and larger number of siblings. Only children had nearly double the prevalence of antihistamines and Epinephrine compared to 4th or later children and around 40–50% higher prevalence for eczema and asthma medications. Among first-borns, a smaller age difference with the second-born sibling was associated with decreased atopic medication prevalence, except for antihistamines and asthma medication of twins (Table 2, panel B).

**Table 2:** Prevalence of childhood atopic medication purchases in ages 0–15 (%) by (A) birth order and number of siblings for the full population and (B) age difference with second-born sibling for first-born children. Birth cohorts 1995–2004

	N	Antihistamines	Eczema medication	Asthma medication	Epinephrine
**A) Birth order (#) by number of children in the family**					
#1/1	88 481	43.1	33.3	20.0	2.8
#1/2	112 893	42.7	33.5	20.1	3.4
#2/2	111 258	38.1	31.6	18.1	2.8
#1/3	46 389	39.3	31.6	18.7	3.3
#2/3	48 000	34.6	29.3	17.1	2.6
#3/3	51 630	33.4	28.4	15.3	2.3
#1/4+	19 113	32.8	28.1	15.6	2.5
#2/4+	19 237	29.5	26.7	15.3	2.2
#3/4+	20 888	27.4	25.8	14.9	2.0
#4+/4+	41 188	23.7	23.9	13.0	1.4
Total	559 077	37.2	30.7	17.8	2.8
**B) First-born’s age difference with second-born**					
Twins	9 237	37.0	28.4	20.6	2.3
1 year	21 650	35.7	30.3	16.9	2.6
2 years	66 300	40.1	32.5	18.6	3.5
3 years	39 629	42.4	32.9	20.1	3.4
4 years	18 831	43.4	33.8	20.2	3.4
5 + years	22 748	43.7	33.7	20.6	3.4
Only child	88 481	43.1	33.3	20.0	2.9
Total	266 876	41.5	37.7	19.5	3.2

### Birth order and family size – *total population analysis*

Children of different birth orders and family sizes differed by observed confounders (Table 3). Only children and first-born children were more likely to be born in urban areas and later-born children in rural areas. Only children, first-borns, and twins in particular, were more likely to be born by Caesarian section. And only children and children in large families were more likely than others to have parents with less than tertiary education and below median income. Except for the lower prevalence in large families, differences in parental atopic medication purchases were modest.

**Table 3:** Proportions (%) of observed confounders by (A) birth order and number of siblings for the full population and (B) age difference with second-born sibling for first-born children. Birth cohorts 1995–2004

	N	Rural residence	Parental tertiary education	Parental above median income	Caesarian section	Parental antihistamines	Parental eczema med.	Parental asthma med.	Parental Epinephrine	
**A) Birth order (#) by number of children**										
#1/1	88 481	24.7	40.3	38.2	22.4	65.2	68.1	46.6	4.6	
#1/2	112 893	25.1	57.8	54.3	20.5	66.7	68.2	46.6	5.1	
#2/2	111 258	27.9	58.1	45.3	15.0	65.7	68.9	46.6	5.0	
#1/3	46 389	28.3	56.9	49.1	16.9	64.8	66.4	44.1	4.9	
#2/3	48 000	31.8	56.8	40.3	13.9	63.7	66.8	44.6	4.7	
#3/3	51 630	36.6	57.4	38.2	14.2	62.7	67.4	44.7	4.6	
#1/4+	19 113	33.4	40.1	31.6	12.7	60.4	63.2	39.6	4.3	
#2/4+	19 237	37.4	46.4	24.9	8.9	60.0	64.1	40.3	4.4	
#3/4+	20 888	41.8	49.1	24.6	11.6	59.6	64.8	40.7	4.5	
#4+/4+	41 188	51.1	45.0	15.3	10.7	56.7	66.3	39.7	4.1	
Total	559 077	30.8	52.9	41.1	16.5	64.0	67.4	44.9	4.8	
**B) First-born’s age difference with second-born**										
Twins	9 237	26.1	57.8	44.3	51.2	66.4	70.0	47.8	5.3	
1 year	21 650	30.3	44.6	42.5	15.2	63.8	66.5	44.6	4.8	
2 years	66 300	27.3	58.3	53.5	16.2	65.3	67.0	44.8	4.9	
3 years	39 629	25.9	59.3	54.0	17.4	66.1	67.2	45.2	5.1	
4 years	18 831	25.5	57.1	51.6	18.4	65.8	67.6	45.4	5.1	
5 + years	22 748	25.3	49.9	44.9	18.9	66.0	67.3	45.6	4.9	
Only child	88 481	24.7	40.3	38.2	22.4	65.2	68.1	46.6	4.6	
Total	266 876	26.1	50.6	46.4	19.9	65.4	67.5	45.7	4.9	

**Fig. 1:**
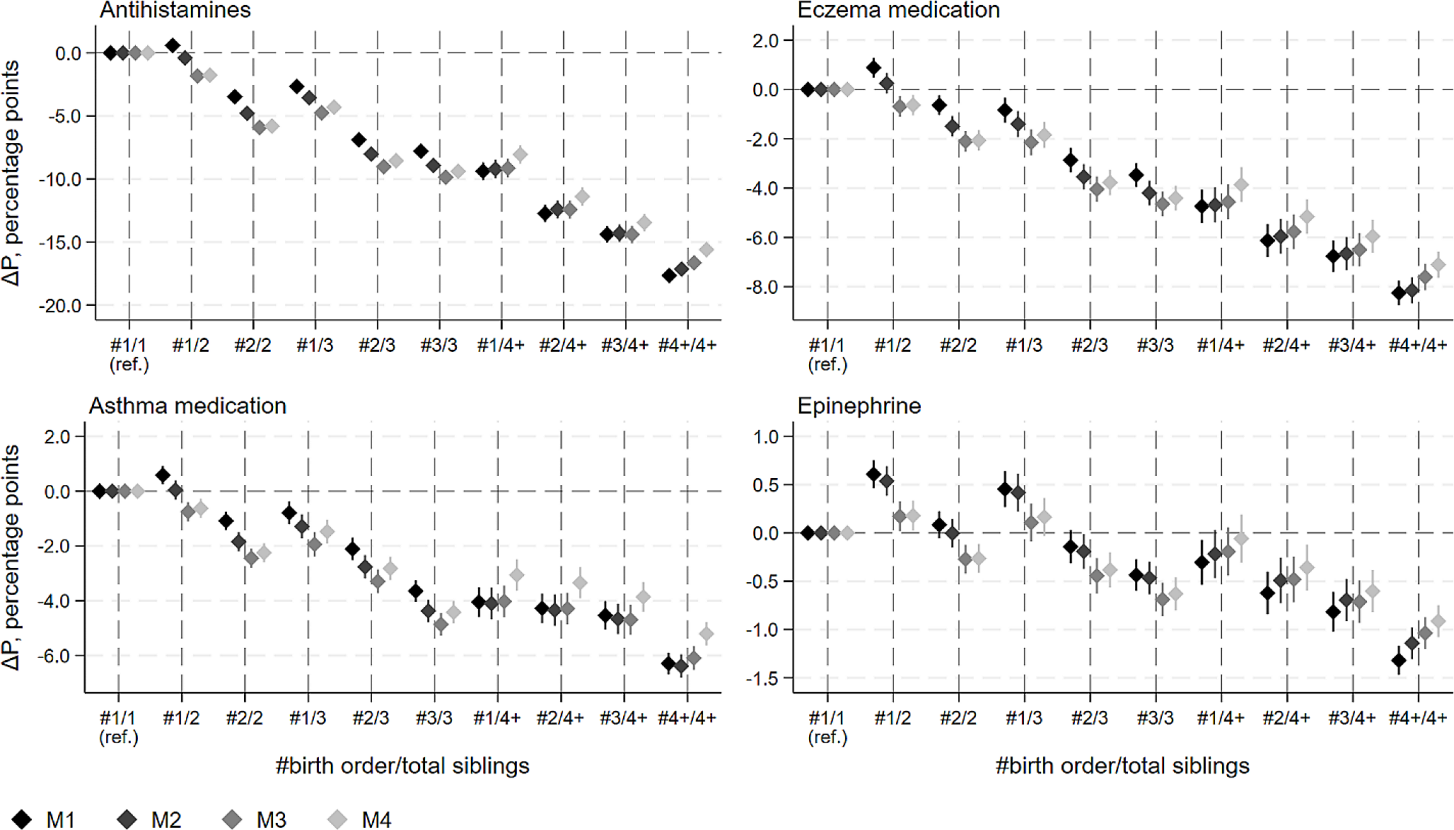
Estimated difference in probability (∆P, percentage points) of childhood atopic medication purchase at ages 0–15 by birth order and number of siblings. Results from total population models with 95% confidence intervals. M1 is adjusted for: child’s sex, birth year, and immigrant background. M2: M1 + region and urbanicity of residence. M3: M2 + household incomeand parental education. M4: M3 + mode of delivery and parental atopic medication purchases

The probability of purchasing atopic medications declined with increasing birth order and family size, and the results were highly similar across medication types (Fig. 1). The absolute percentage-point decreases were largest for antihistamines and smallest for Epinephrine, a much less commonly used medication type, and thus also the scales in Fig. 1 vary across medication types. Notably, there was a clear decrease in probability by birth order even within each family size category, indicating that the association of atopic medication purchases with birth order is not merely due to the fact that higher birth order children come from larger families.

The differences were accentuated when controlling for the urban residence (Model 2) and lower socioeconomic position (Model 3) of one-child families but attenuated when additionally controlling for mode of delivery and parental atopic medication use (Model 4). In this final model, the differences in antihistamine, eczema, asthma and Epinephrine medication use between e.g. the third child of a three-child family and an only child were 9.4 (95% CI; -9.9; -8.9), 4.4 (95% CI; -4.9; -3.9), 4.4 (95% CI; -4.8; -4.0) and 0.6 (95% CI; -0.8; -0.5) percentage points, respectively. Relative to the overall prevalence of each medication type (Table 1, panel A), these reflect around 15–25% differences in prevalence.

### Birth order – sibling population analysis

In Table 4 we assessed birth order among children with siblings to assess the importance of measured and unmeasured familial confounding. Controlling for age, sex and immigration background (Model 1a) the risk for atopic medication purchases relative to the overall prevalence of each medication type (Table 2, panel A) was around 10–20% lower among second-born children, around 20–40% percent lower among third-born children and around 30–70% lower among fourth and subsequent children compared to first-borns. Around 25–45% of the lower atopy risk among birth orders three and higher was due to larger family size, controlled for in Model 1b. The lower atopy risk of second born children, however, was not explained by family size.

Further controlling for other observed confounders in Models 2–4, and all unobserved exposures shared by siblings in the fixed effects Model 5, had little impact on the estimates for antihistamines and Epinephrine. However, the lower risk for eczema and asthma medication purchases in higher birth orders was mostly explained by unobserved family confounding, with a less than 10% reduction in risk remaining in the full model even among fourth and higher order children (Model 5).

**Table 4:** Estimated difference in probability (percentage points) of childhood atopic medication purchases by birth order. Comparison of OLS models (M1–M4) and sibling fixed effects models (M5) with 95% confidence intervals. (Sibling-population *N* = 324,306)

	Birth order (ref. 1st)			Birth order (ref. 1st)		
	2nd	3rd	4th +	2nd	3rd	4th +
	**Antihistamines**			**Eczema medication**		
M1a	-5.1 (-5.5; -4.7)	-10.7 (-11.3; -10.2)	-18.7 (-19.3; -18.1)	-2.4 (-2.8; -2.0)	-5.3 (-5.9; -4.8)	-9.3 (-9.8; -8.7)
M1b	-4.6 (-5.0; -4.2)	-6.9 (-7.5; -6.3)	-11.7 (-12.4; -11.0)	-2.0 (-2.4; -1.7)	-3.1 (-3.6; -2.5)	-5.3 (-6.0; -4.6)
M2	-4.4 (-4.8; -4.0)	-6.6 (-7.2; -6.0)	-10.4 (-11.1; -9.7)	-1.9 (-2.3; -1.6)	-2.9 (-3.4; -2.3)	-4.7 (-5.4; -4.0)
M3	-4.2 (-4.6; -3.8)	-6.4 (-6.9; -5.8)	-9.7 (-10.4; -9.0)	-1.7 (-2.1; -1.3)	-2.6 (-3.1; -2.0)	-4.0 (-4.7; -3.3)
M4	-4.2 (-4.6; -3.8)	-6.4 (-6.9; -5.8)	-9.5 (-10.2; -8.8)	-1.7 (-2.1; -1.3)	-2.6 (-3.2; -2.1)	-4.1 (-4.8; -3.4)
M5	-4.9 (-5.5; -4.4)	-7.8 (-8.8; -6.7)	-10.5 (-12.2; -8.8)	-1.6 (-2.1; -1.0)	-2.0 (-3.0; -0.9)	-2.4 (-4.1; -0.7)
	**Asthma medication**			**Epinephrine**		
M1a	-1.4 (-1.8; -1.1)	-4.0 (-4.4; -3.6)	-6.3 (-6.7; -5.8)	-0.5 (-0.7; -0.4)	-1.2 (-1.3; -1.0)	-1.9 (-2.1; -1.8)
M1b	-1.2 (-1.6; -0.9)	-2.6 (-3.1; -2.2)	-4.0 (-4.5; -3.4)	-0.5 (-0.6; -0.4)	-0.9 (-1.1; -0.7)	-1.4 (-1.6; -1.2)
M2	-1.2 (-1.5; -0.9)	-2.5 (-3.0; -2.0)	-3.6 (-4.1; -3.0)	-0.5 (-0.6; -0.3)	-0.9 (-1.1; -0.7)	-1.2 (-1.5; -1.0)
M3	-1.1 (-1.4; -0.7)	-2.4 (-2.8; -1.9)	-3.2 (-3.7; -2.6)	-0.4 (-0.5; -0.2)	-0.8 (-1.0; -0.6)	-1.1 (-1.3; -0.8)
M4	-1.0 (-1.4; -0.7)	-2.4 (-2.9; -1.9)	-3.1 (-3.7; -2.5)	-0.4 (-0.5; -0.2)	-0.8 (-1.0; -0.6)	-1.1 (-1.3; -0.8)
M5	-0.5 (-1.0; -0.1)	-1.0 (-1.8; -0.2)	-1.5 (-2.8; -0.2)	-0.5 (-0.7; -0.3)	-1.1 (-1.4; -0.7)	-1.6 (-2.1; -1.0)

Abbreviations; ref. = reference category

M1a adjusted for: child’s sex, birth year, and immigrant background. M1b: M1a + number of siblings. M2: M1 + region and urbanicity of residence

M3: M2 + household income and parental education. M4: M3 + mode of delivery and parental atopic medication purchases. M5: M4 + sibling fixed effects

### Age difference – analysis of first-borns

**Fig. 2:**
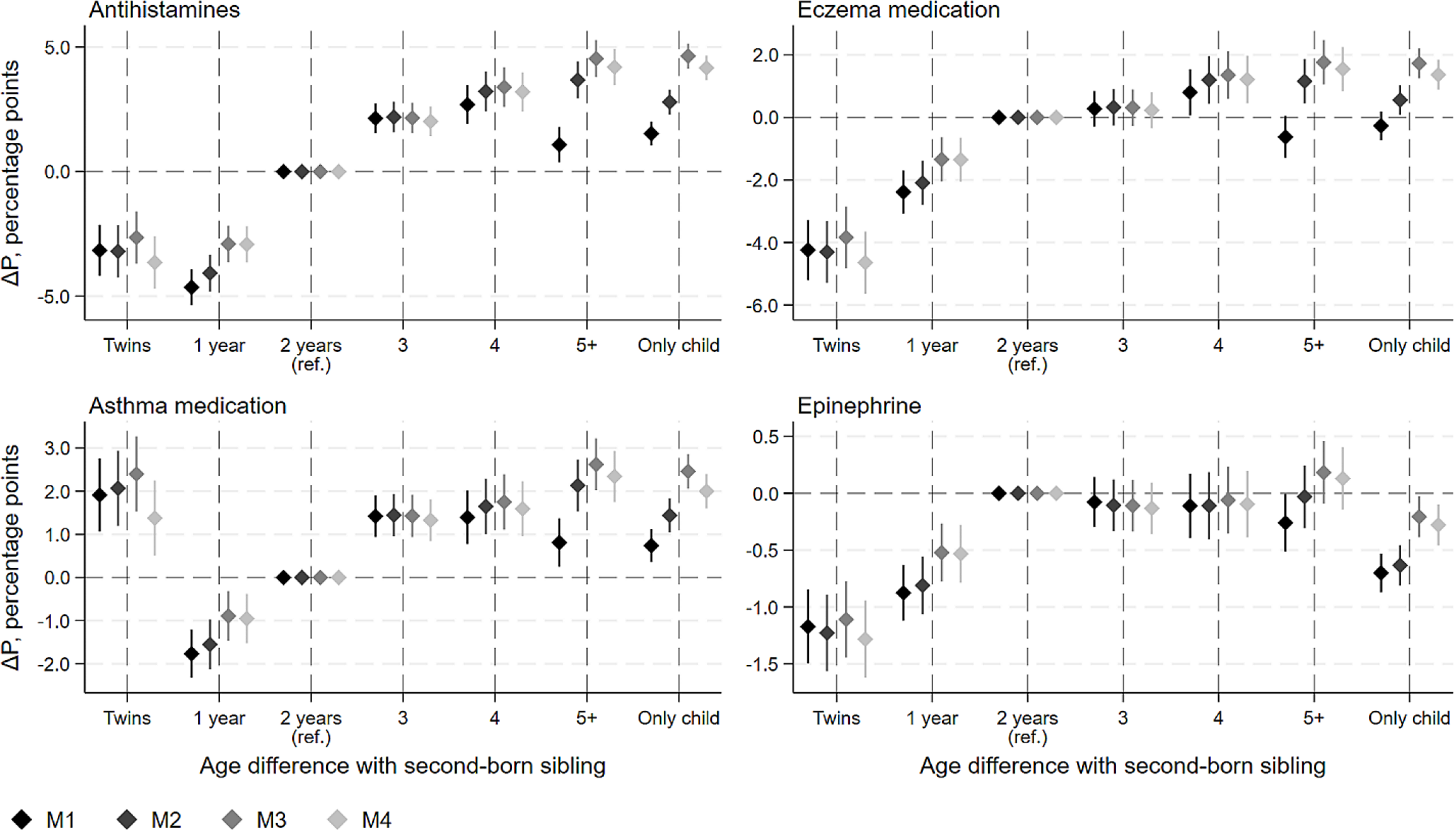
First-born child’s estimated difference in probability (∆P, percentage points) of childhood atopic medication purchase at ages 0–15 by age difference with second-born sibling (years) with 95% confidence intervals. M1 adjusted for: child’s sex, birth year, and immigrant background. M2: M1 + region and urbanicity of residence. M3: M2 + household income and parental education. M4: M3 + mode of delivery and parental atopic medication purchases

Among first-born children, the smaller the age difference with the second-born sibling, the lower the likelihood of purchasing atopic medication, although the difference no longer increased for age differences above three years (Fig. 2). For example, compared with the median age difference of two years, a one-year age difference was related to a reduction of 2.9 p.p (95% CI; -3.6; -2.2) in the probability of purchasing antihistamines in the final model. Relative to the prevalence of each medication type among first-borns (Table 1, panel B), the estimates reflect 5–15% decreases across medication types for those with only a one-year difference to the second born. In general, the risk for atopic medication purchases was smallest among twins and largest among only children. However, there was an increased risk for asthma medication use among twins and a decreased risk for Epinephrine use among only children.

### Sensitivity analyses

The results from models restricted to families where the children lived in the same household with both of their biological parents for the whole observation period without any half- or step-siblings were highly similar to the full population models (Supplementary Figures S1 and S2). The results from models assessing purchases in more than one, two, or three years by age 15 were similar but weaker with the higher cut-points (Supplementary Figures S3 and S4).

The results of the age difference models remained largely unchanged when controlling for unobserved familial confounding using cousin fixed effects models, although the confidence intervals were wider due to the restricted sample (Supplementary Figure S5). The results for additional analyses on the age difference between second-born and third-born children resembled those of the first-born and second-born children, but the differences were slightly smaller and less often significant, likely because an older sibling already exists and three-sibling families are fewer (Supplementary Figure S6).

The results regarding special reimbursement rights to asthma medication by both birth order and age difference between first- and second-born siblings (Supplementary Figure S7) were similar to those with asthma medication purchases.

We further investigated whether children with higher genetic or environmental risk for atopic diseases, as indicated by having a parent with atopic medication use or living in urban environments, could be particularly sensitive to sibling exposures. Overall, there was little modification in the sibling effects by parental atopic medication use (any vs. no atopic medication purchases between offspring ages 0–15) or urban-rural residence (born rural vs. other). However, the birth-order effects were somewhat stronger among children from urban environments and with parental atopic diseases (Supplementary Figures S7–S11).

## Discussion

While the inverse relationship between having older siblings and developing atopic diseases is well established [6, 7], important confounders have largely been neglected in prior studies. Using population data on over 500 000 children nested in families, we showed that while unobserved environmental and genetic familial confounding indeed explained much of the lower risk among children with a higher birth order, particularly for eczema and asthma medication, higher birth order still remained strongly protective.

While we cannot rule out that the birth order effects we observed could reflect parity-related differences in in-utero exposures, we also showed that among first-born children, a smaller age difference to the closest younger sibling was an important predictor of decreased atopic medication purchases. In fact, having no age difference, i.e. having a twin, was most protective against atopic diseases other than asthma. Our results are in line with recent findings of a lower age difference between siblings being an important predictor of richer airway and gut microbiota [10], and suggest that first-born children benefit from having younger siblings early in life. Taken together, our findings strongly imply that exposure to siblings, particularly very early in life, truly contributes to the post-natal environment and decreases the likelihood of developing atopic diseases. With regard to eczema and asthma, for which familial confounding explained a large part of the differences by birth order, further research is needed to fully understand the specific mechanisms that explain the lower risk among younger siblings.

The associations between birth order and antihistamine and Epinephrine use were sizeable and likely to have clinical relevance. Even after controlling for all exposures shared by siblings, the probability of antihistamine use was around 20% lower and the probability of Epinephrine use around 40% lower among third-born than first-born children, and around 30–60% lower among subsequent children. These effects are of a similar magnitude to the well established ‘farm effect’ showing that children growing up on farms are around 30–80% less likely than others to develop atopic diseases [27, 28]. Our results indicate that benefits similar to those provided by the farm environment can also be obtained by exposures brought about by siblings.

Although we found support for the microbiota hypothesis by assessing the exposure provided by coresident siblings, these beneficial exposures need not necessarily stem from within the family. For example, the results of a Finnish sandbox study encouragingly show that an intervention enriching the microbiota of kindergarten playgrounds increased the commensal microbiota among day care children [29], which potentially has huge preventive implications regarding the development of atopic diseases. The implication of our study – and others – is that environmental microbiota should be taken into consideration when planning interventions to reduce atopic diseases.

### Strengths and limitations

A major strength of this study is the unique longitudinal population register data with linkages between family members that allowed us to assess observed and unobserved familial confounding in a manner that has not been done before. However, there are also limitations that need to be acknowledged.

The reimbursed prescription medication purchases captured by our data may not fully reflect the existence and severity of atopic diseases. Some atopic medication products are available over the counter and some are otherwise not reimbursed and thus not covered by our data. Consequently, children with atopic symptoms, but who use non-reimbursed medications or are not treated, were misclassified as having no symptoms. This may lead to an underestimation of atopic diseases in the population. However, our asthma medication measure (inhaled corticosteroids and combination inhalers) has been found to be highly accurate in identifying diagnosed asthma [30], and the results from our sensitivity analyses for the special reimbursement right for asthma medication based on diagnosis were highly similar to those observed for the other medication types.

The underestimation of the level of atopic diseases may also introduce bias in our birth order and family size estimates in the full population if medication use patterns are correlated with family size. However, as the results in sibling comparisons that control for between-family differences in treatment seeking patterns were highly similar to the full population results, this bias is likely to be small. Furthermore, the patterns of results were similar for a wide variety of medication types for diseases ranging from allergic rhinitis to potentially life-endangering allergic reactions, providing further validity to our results.

Our sibling fixed effects models control for everything that is shared between siblings, however, residual confounding might occur if there are systematic environmental changes correlated with birth order, such as moving to greener and more biodiverse environment as the family grows larger. However, this bias appears unlikely as the sibling differences in the characteristics of the place of residence at time of birth were controlled for in the within-family fixed effects models.

Finally, with our fully register-based data, we had no direct measures for microbiota exposure and thus could not directly test the pathways from sibling exposures to atopic diseases via microbial exposure. For such analyses, linking microbial samples from individuals to healthcare and medication records could be a fruitful way forward in future studies.

## Conclusions

Using full population data with extensive controls for observed and unobserved environmental and genetic confounding, our study provides strong evidence that, while confounding does play an important role, having older or younger siblings in early childhood protects from atopic diseases – thus giving support to the so-called microbiota hypothesis. Our results imply the need for preventive interventions in which all children would benefit from the same kind of diverse microbiota exposure as children in large families.

### Electronic supplementary material

Below is the link to the electronic supplementary material.

Supplementary Material 1
