# Supplementary material for: Does having siblings really protect against childhood atopic diseases? A total population and within-family analysis
Source: Eur J Epidemiol. 2024 Feb 6;39(3):289–98. doi: 10.1007/s10654-024-01104-w (PMC10995035; doi:10.1007/s10654-024-01104-w)
Supplement: Supplementary file 1 — Supplementary Material 1 [file 10654_2024_1104_MOESM1_ESM.docx]

**Supplementary Fig. S1**

**
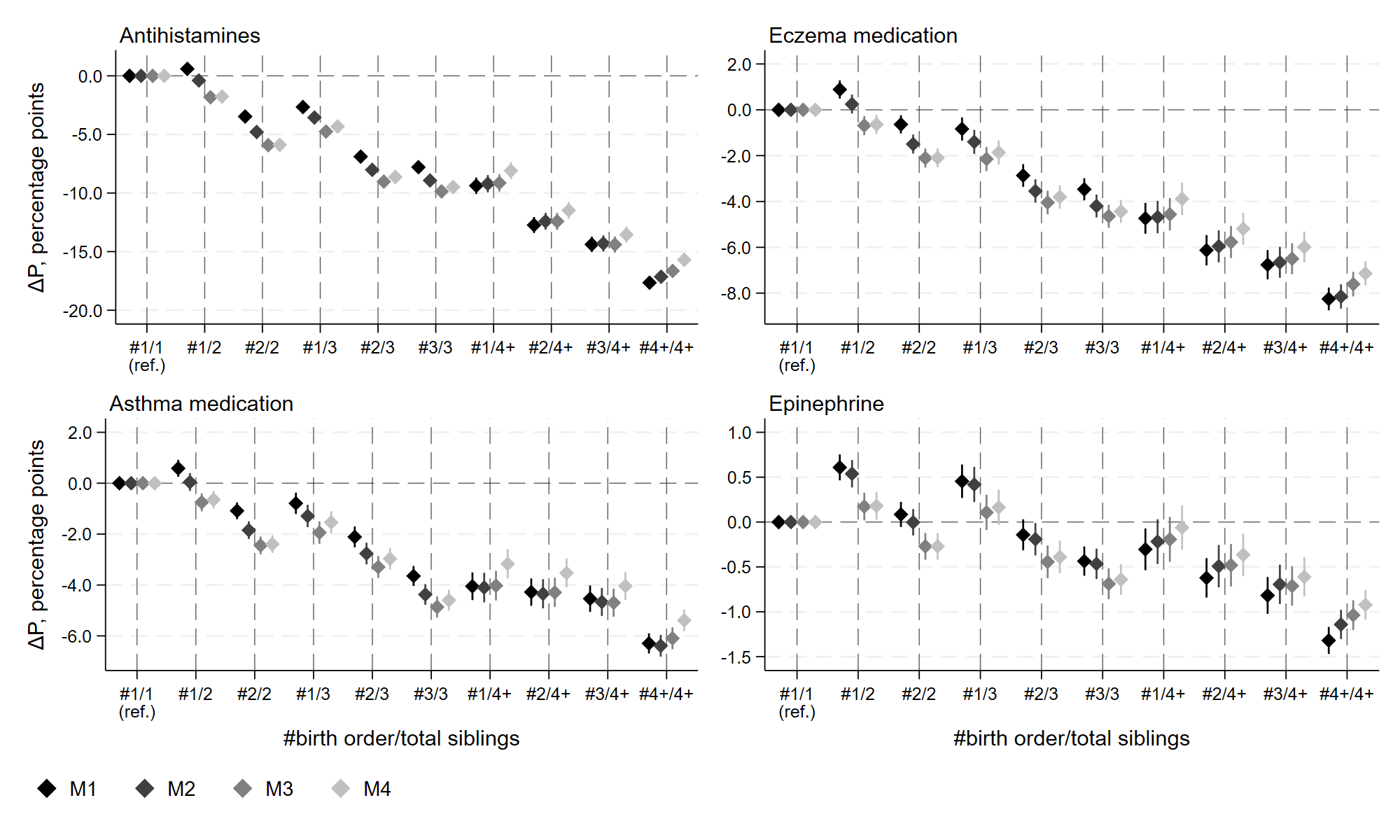
**

Estimated difference in probability (∆P, percentage points) of childhood atopic medication purchase at ages 0–15 by number of siblings and birth order among them. Results from fully adjusted models with 95% confidence intervals. Sample limited to children without half- or stepsiblings and whose biological parents lived in the same household for the entire observation period (N=284,187). M1 is adjusted for: child’s sex, birth year, and immigrant background. M2: M1+region and urbanicity of residence. M3: M2+household income and parental education. M4: M3+mode of delivery and parental atopic medication purchases.

**Supplementary Fig. S2**

**
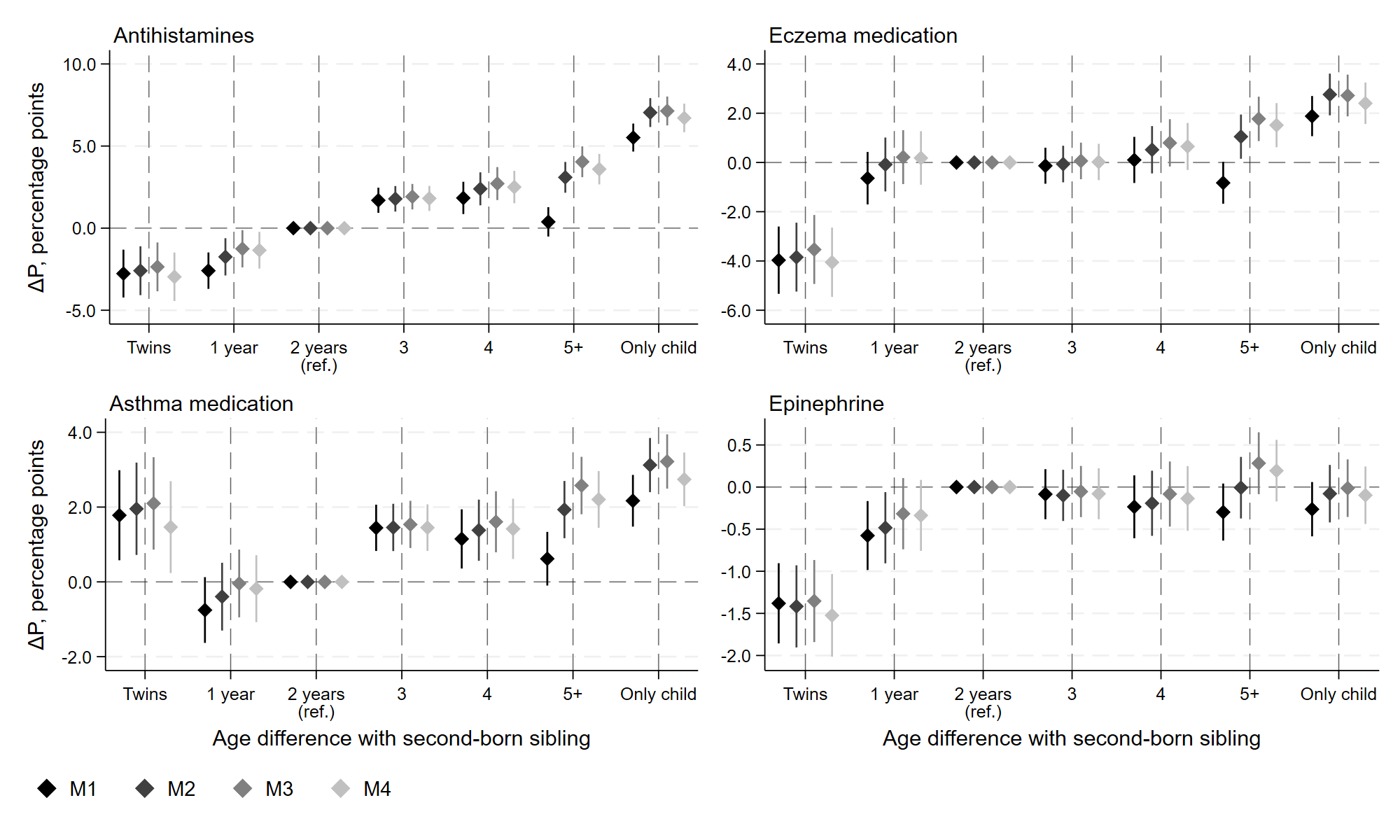
**

First-born child’s estimated difference in probability (∆P, percentage points) of childhood atopic medication at ages 0–15 by age difference with second-born sibling (years) with 95% confidence intervals. Sample limited to children without half- or stepsiblings and whose biological parents lived in the same household for the entire observation period (N=129,178). M1 is adjusted for: child’s sex, birth year, and immigrant background. M2: M1+region and urbanicity of residence. M3: M2+household income and parental education. M4:M3+mode of delivery and parental atopic medication purchases.

**Supplementary Fig. S3**

**
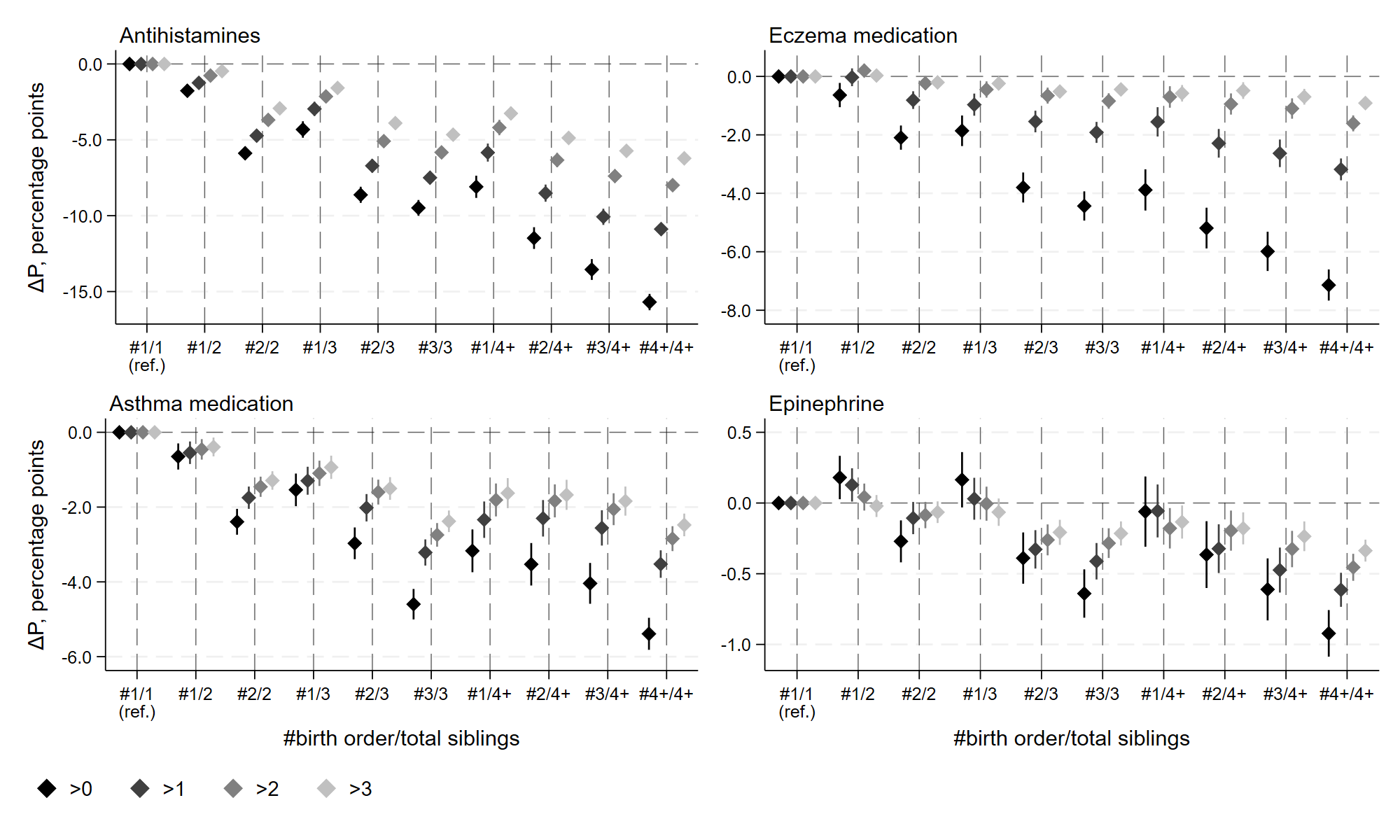
**

Estimated difference in probability (∆P, percentage points) of childhood atopic medication purchases at ages 0–15 by number of siblings and birth order among them. Intensity of medication purchases measured with purchase years (>0; >1; >2; >3). Results from fully adjusted total population models with 95% confidence intervals (N=559,077). All models are adjusted for child’s sex, birth year and immigrant background, geographical area and urbanicity, household income and parental education, mode of delivery and parental atopic medication purchases.

**Supplementary Fig. S4**


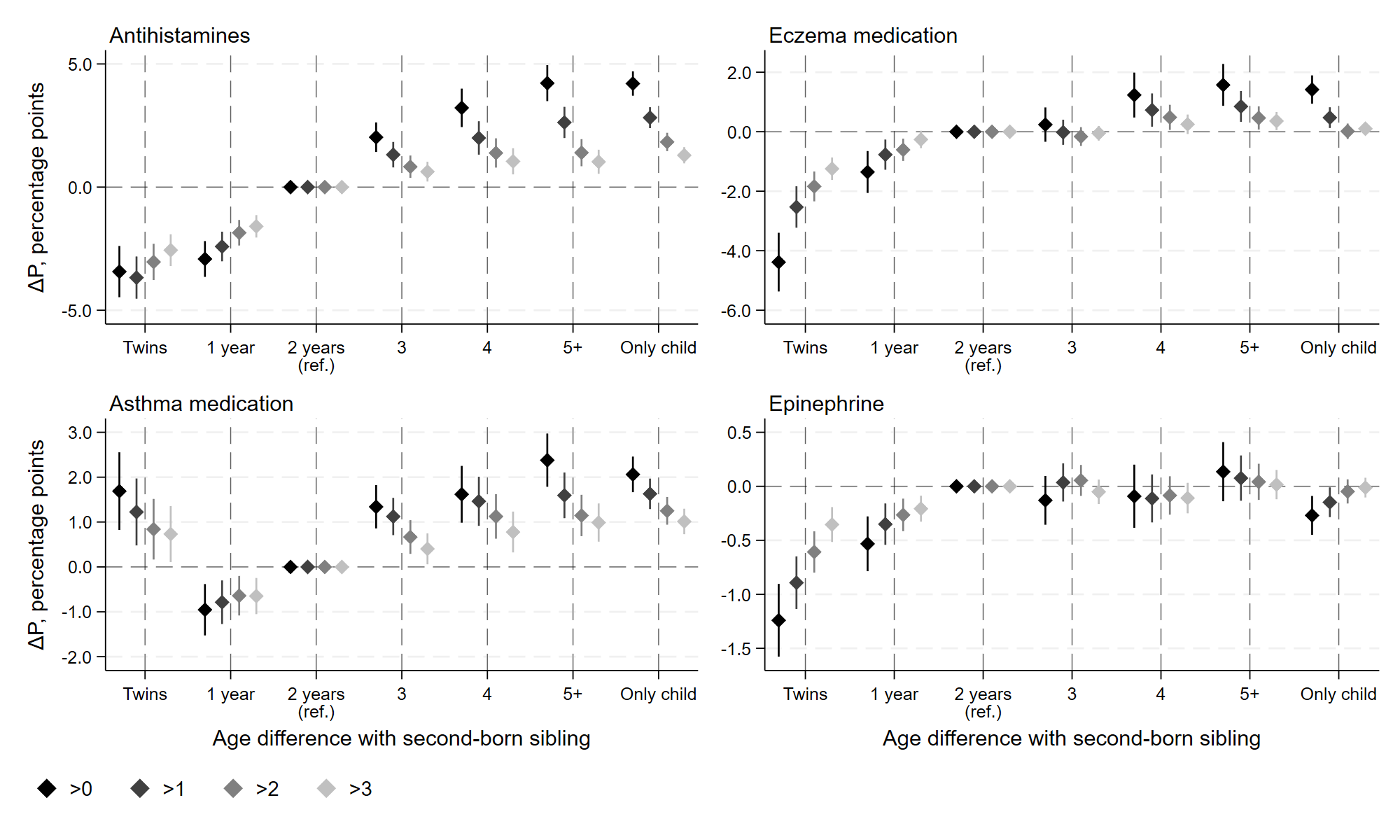


First-born child’s estimated difference in probability (∆P, percentage points) of childhood atopic medication purchase at ages 0–15 by age difference with second-born sibling (years). Intensity of medication purchases measured with purchase years (>0; >1; >2; >3). Results from fully adjusted models with 95% confidence intervals (N=266,876). All models are adjusted for child’s sex, birth year and immigrant background, geographical area and urbanicity, household income and parental education, mode of delivery and parental atopic medication purchases.

**Supplementary Fig. S5**


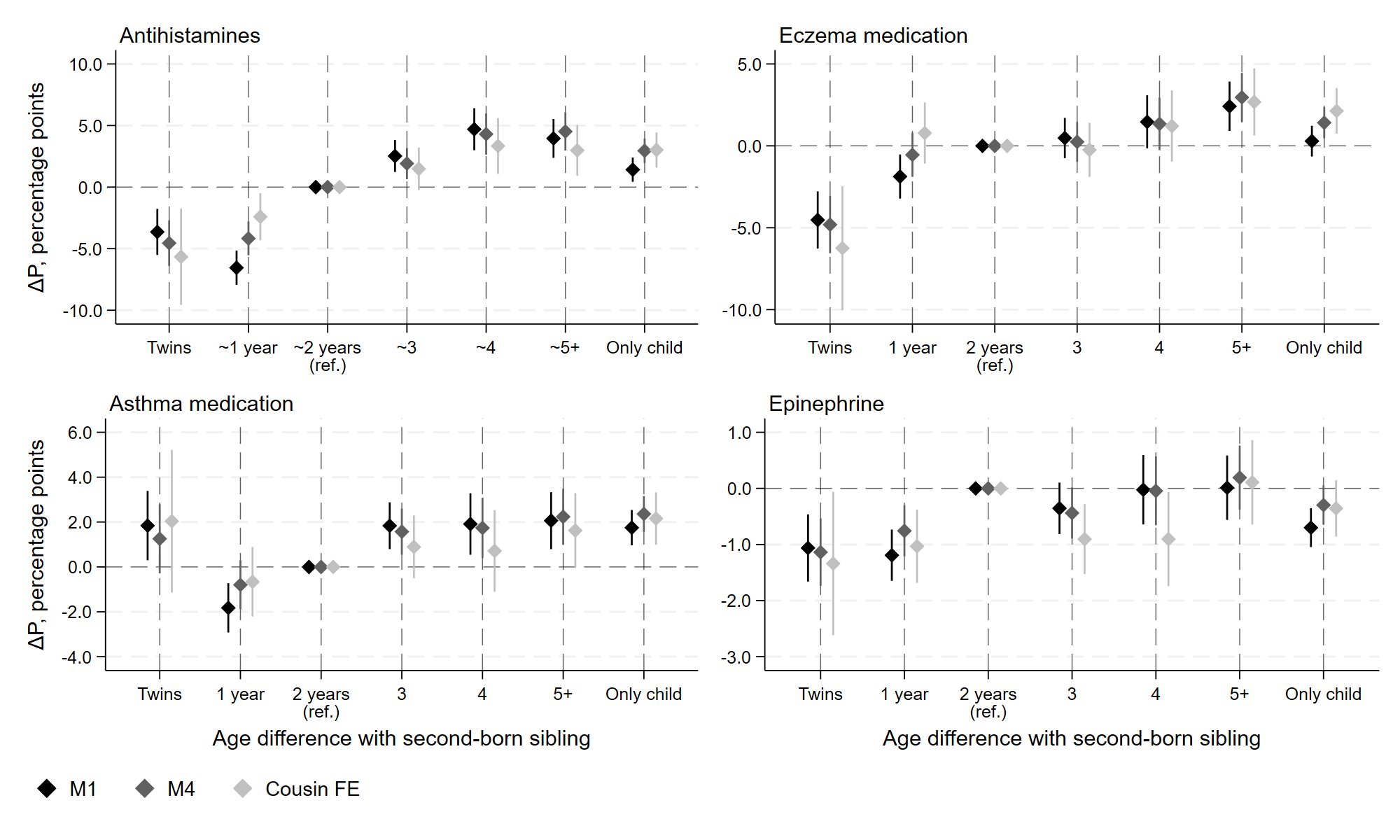


First-born child’s estimated difference in probability (∆P, percentage points) of childhood atopic medication purchase at ages 0–15 by age difference with second-born sibling (years). Results from maternal cousin-population models with 95% confidence intervals (N=66,040). M1 is adjusted for: child’s sex, birth year, and immigrant background. M4: M1+region and urbanicity of residence, household income, parental education, mode of delivery and parental atopic medication purchases. Cousin FE: M4 with cousin fixed effects.

**Supplementary Fig. S6**


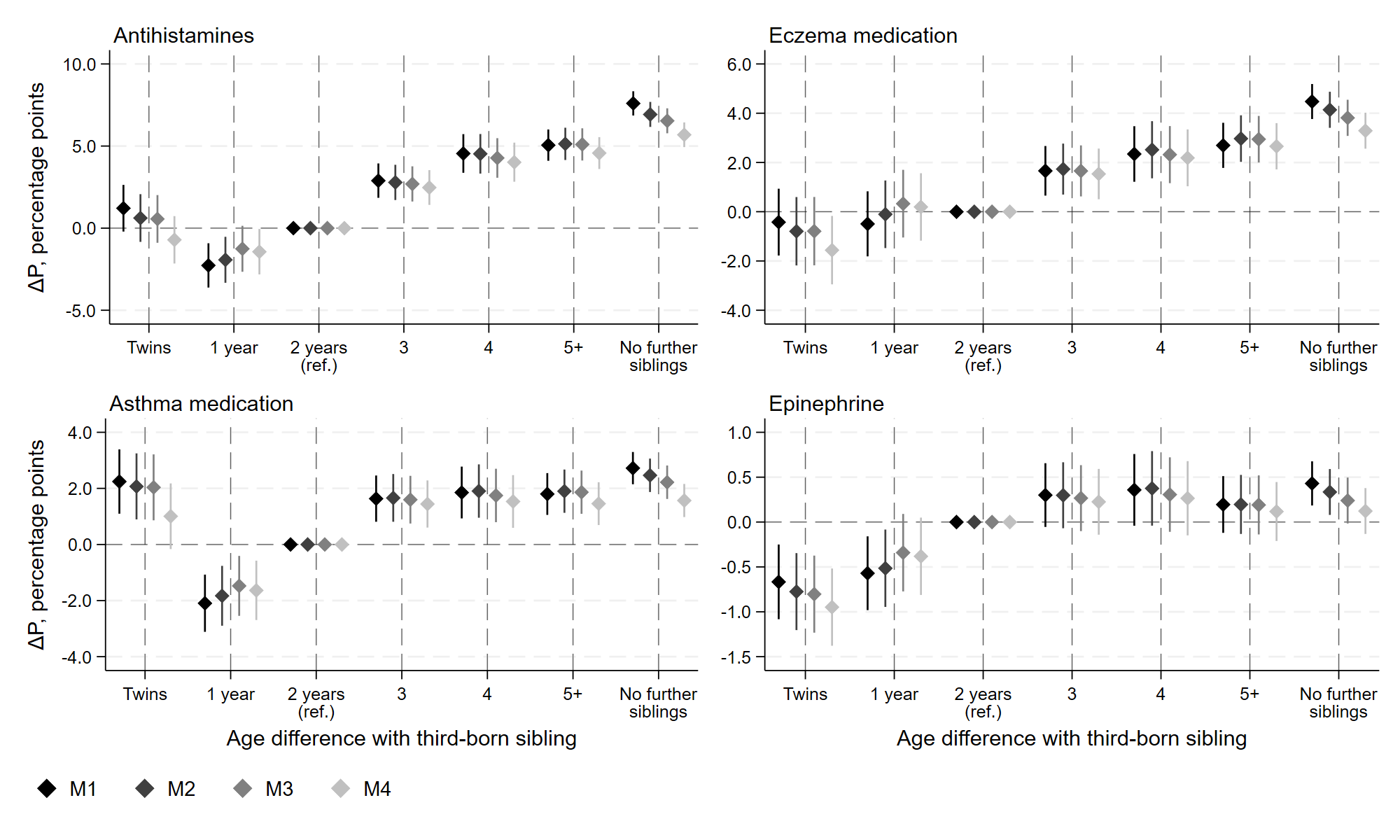


Second-born child’s estimated difference in probability (∆P, percentage points) of childhood atopic medication purchase at ages 0–15 by age difference with third-born sibling (years) with 95% confidence intervals (N=185,867). M1 is adjusted for: child’s sex, birth year, and immigrant background. M2: M1+region and urbanicity of residence. M3: M2+household income and parental education. M4: M3+mode of delivery and parental atopic medication purchases.

**Supplementary Fig. S7**

**
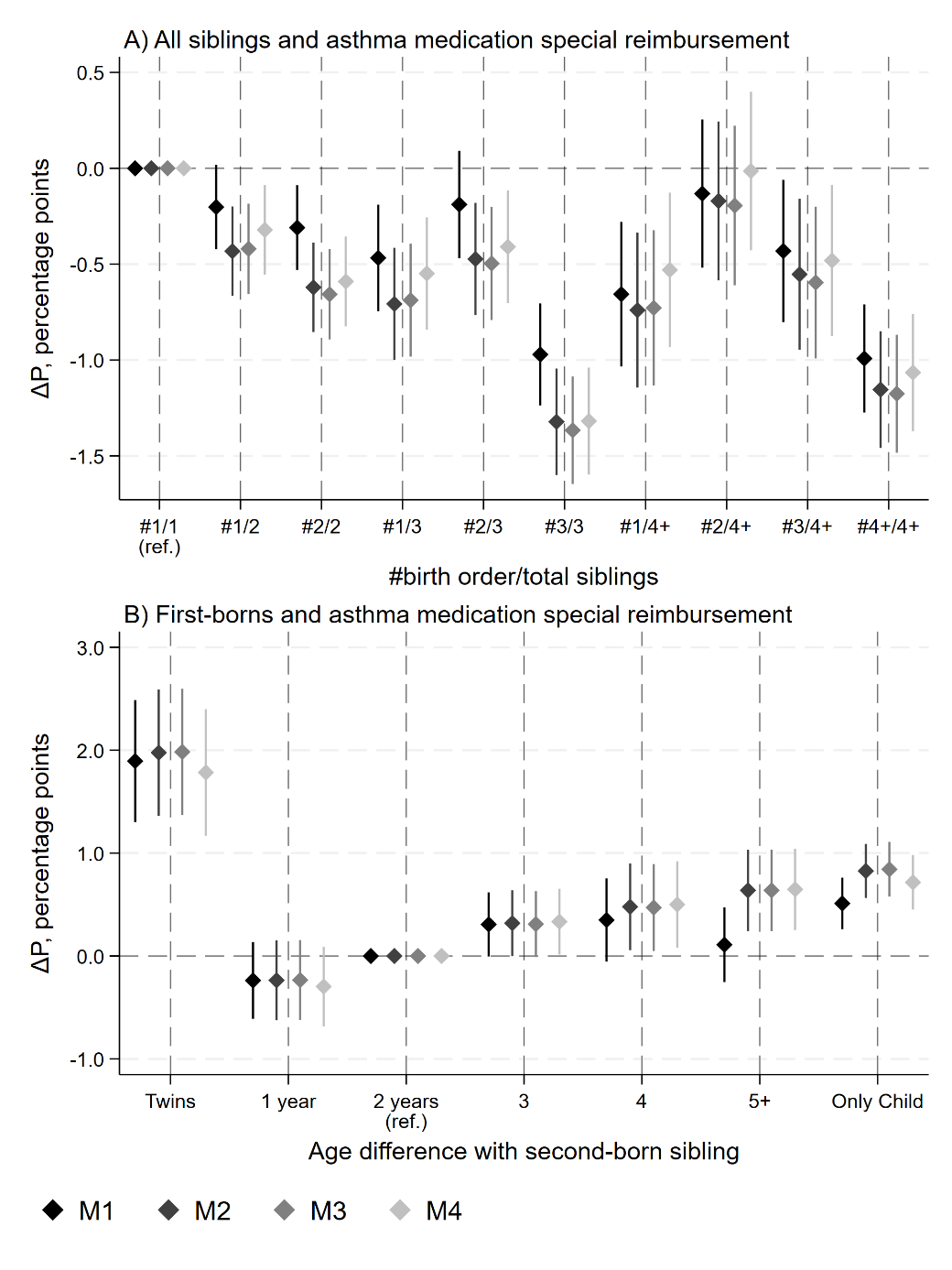
**

Estimated difference in probability (∆P, percentage points) of childhood asthma medication special reimbursement right by A) number of siblings and birth order among them and B) first-born child’s age difference with second-born sibling (years). Fully adjusted models with 95% confidence intervals (A) N=559,077; B) N=266,876). M1 is adjusted for: child’s sex, birth year, and immigrant background. M2: M1+region and urbanicity of residence. M3: M2+household income and parental education. M4: M3+mode of delivery and parental atopic medication purchases.

**Supplementary Fig. S8**

**
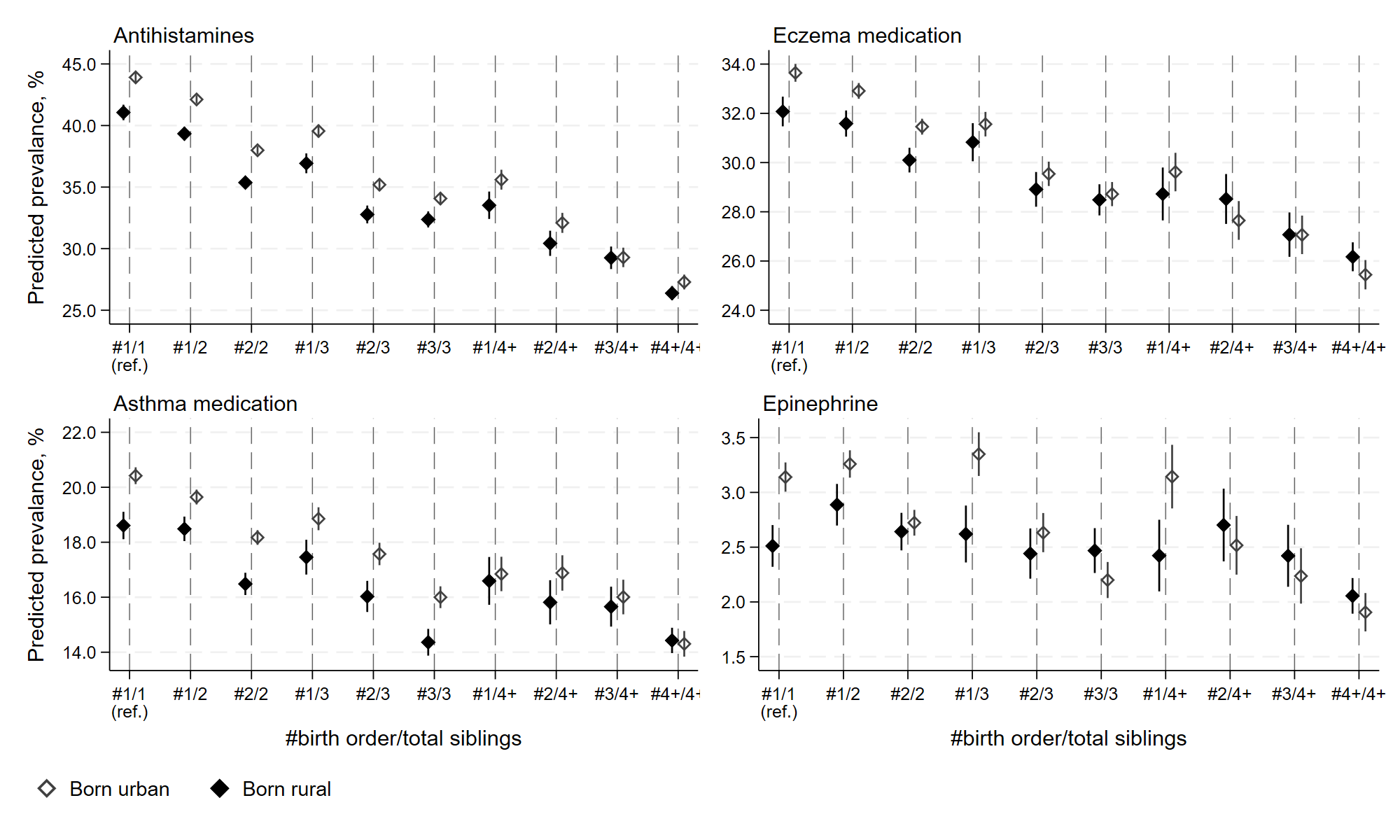
**

Moderation by urban vs. rural birth environment. Estimated prevalences (%) of childhood atopic medication purchase at ages 0–15 by number of siblings and birth order among them. Results from fully adjusted total population models with 95% confidence intervals (N=559,077). Models adjusted for child’s sex, birth year, immigrant background, region, urbanicity of residence, household income, parental education, mode of delivery and parental atopic medication purchases.

**Supplementary Fig. S9**

**
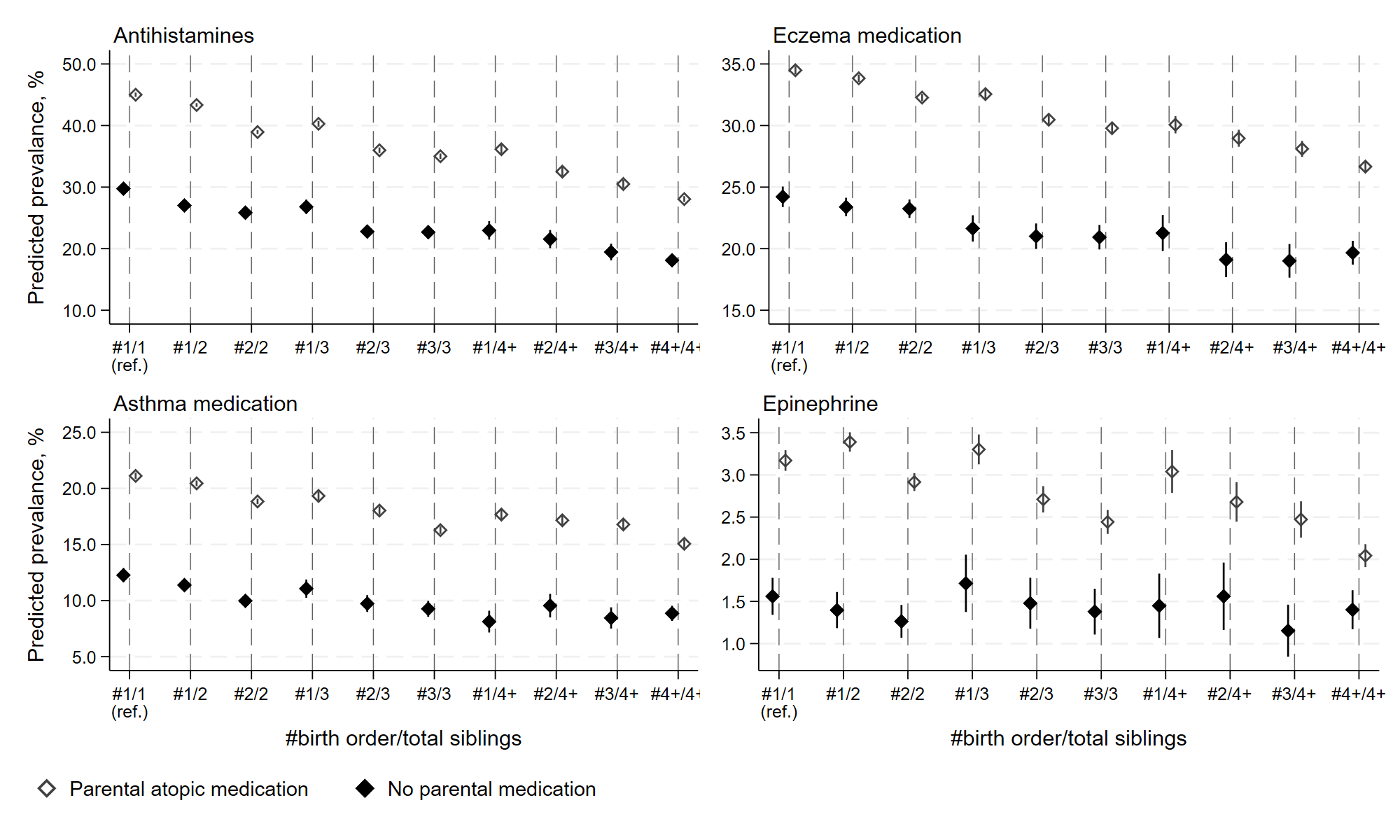
**

Moderation by parental atopic medication use. Estimated prevalences (%) of childhood atopic medication purchase at ages 0–15 by number of siblings and birth order among them. Results from fully adjusted total population models with 95% confidence intervals (N=559,077). Models adjusted for child’s sex, birth year, immigrant background, region, urbanicity of residence, household income, parental education, mode of delivery and parental atopic medication purchases.

**Supplementary Fig. S10**

**
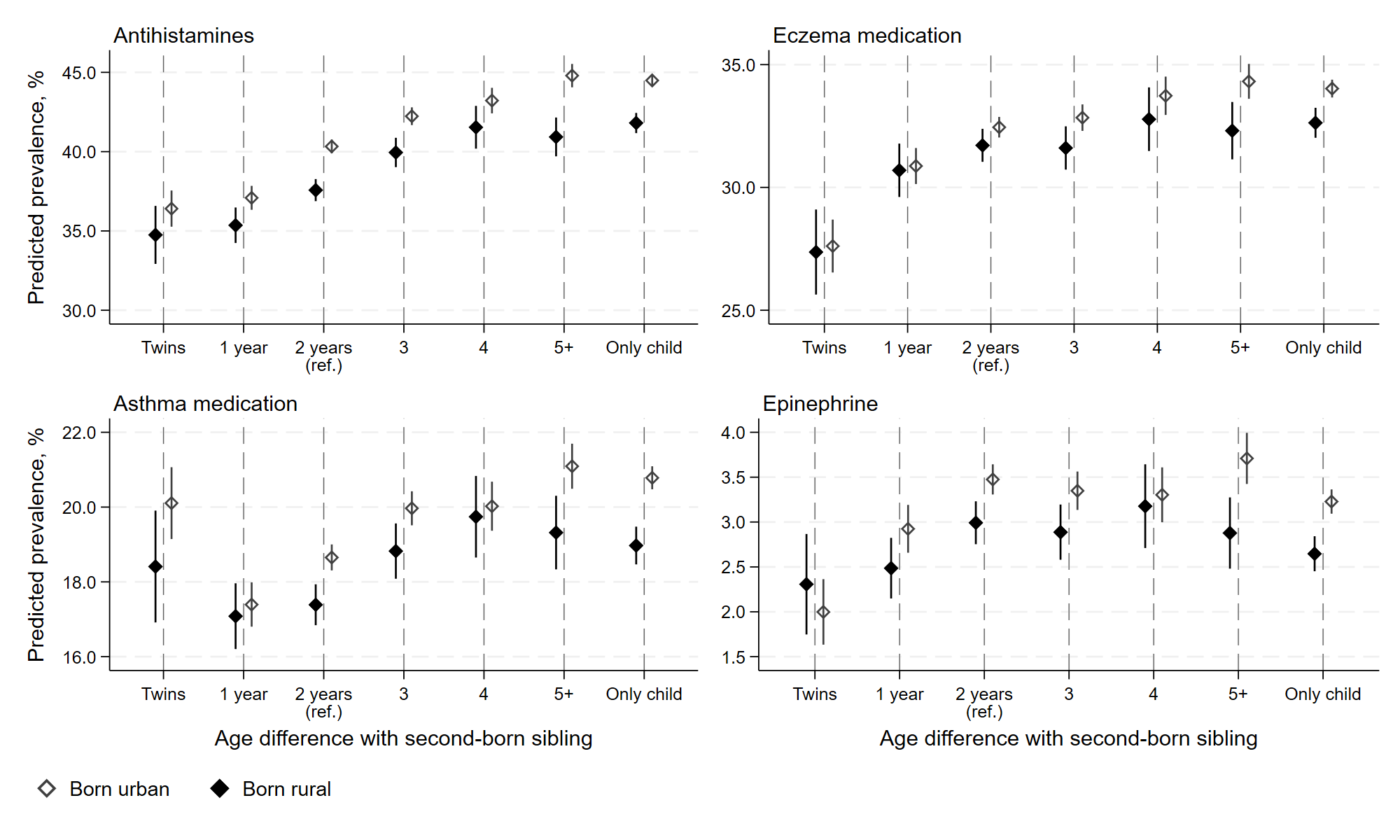
**

Moderation by urban vs. rural birth environment. First-born child’s estimated prevalences (%) of childhood atopic medication at ages 0–15 by age difference with second-born sibling (years). Results from fully adjusted models with 95% confidence intervals (N=266,876). Models adjusted for child’s sex, birth year, immigrant background, region, urbanicity of residence, household income, parental education, mode of delivery and parental atopic medication purchases.

**Supplementary Fig. S11**


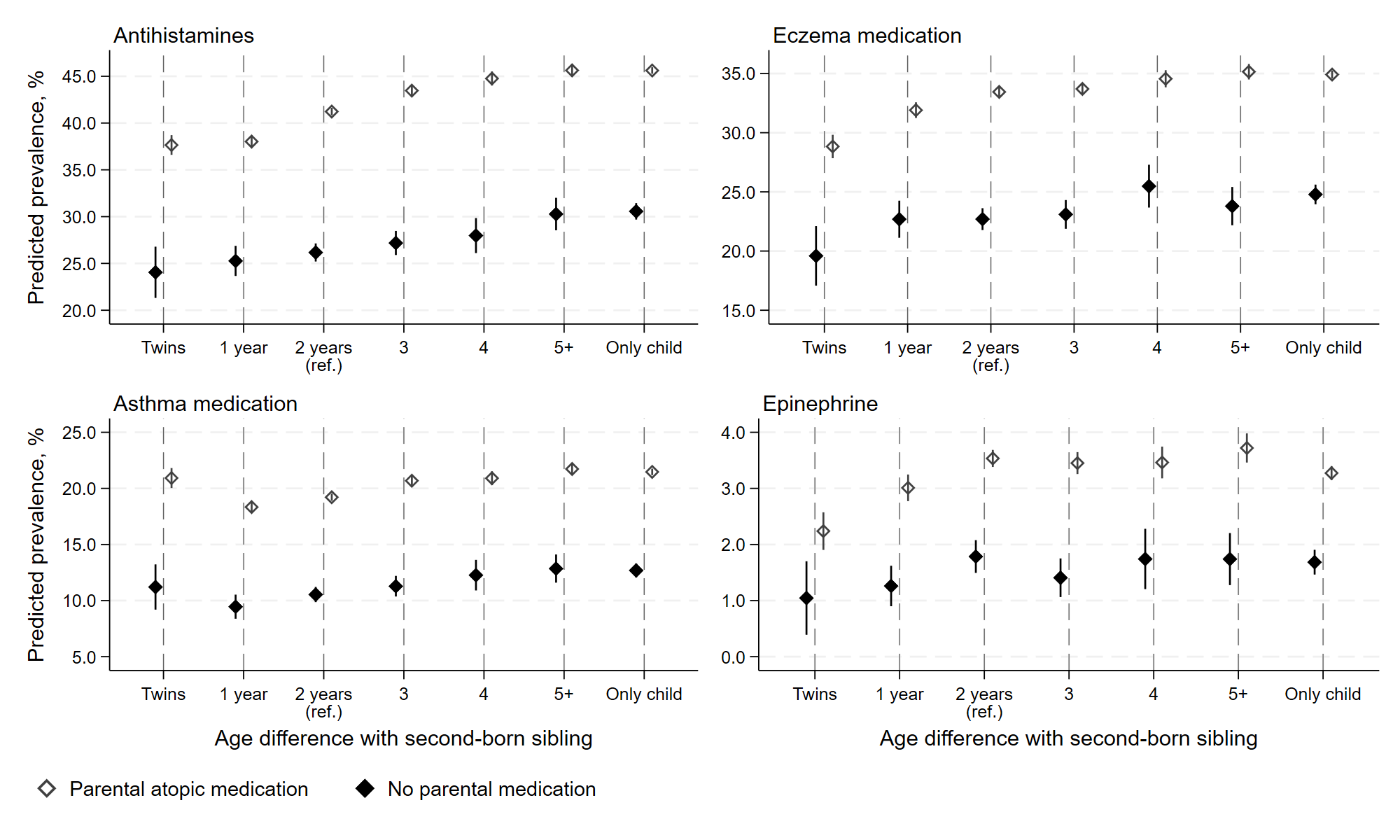


Moderation by parental atopic medication use. First-born child’s estimated prevalences (%) of childhood atopic medication at ages 0–15 by age difference with second-born sibling (years). Results from fully adjusted models with 95% confidence intervals (N=266,876). Models adjusted for child’s sex, birth year, immigrant background, region, urbanicity of residence, household income, parental education, mode of delivery and parental atopic medication purchases.
